# Microeukaryotic habitat specialists exhibit stronger determinism and biodiversity-nutrient cycling relationship than generalists in a subtropical river

**DOI:** 10.1128/aem.01364-25

**Published:** 2025-09-23

**Authors:** Lu Li, Huihuang Chen, Weidong Chen, Jun Yang

**Affiliations:** 1 Key Laboratory for Humid Subtropical Eco-geographical Processes of the Ministry of Education, School of Geographical Sciences, Fujian Normal University 731375, Fuzhou, China; 2 Aquatic EcoHealth Group, Fujian Key Laboratory of Watershed Ecology, State Key Laboratory of Regional and Urban Ecology, Institute of Urban Environment, Chinese Academy of Sciences 85406 , Xiamen, China; University of Delaware, Lewes, Delaware, USA

**Keywords:** generalists, specialists, community assembly, co-occurrence network, multi-nutrient cycling

## Abstract

Microeukaryotic generalists and specialists exhibit varying levels of environmental sensitivity, which contribute differently to community dynamics and are important for aquatic ecosystem functioning. However, the community assembly, co-occurrence network, and impacts of microeukaryotic generalists and specialists' diversity on ecosystem functions remain poorly understood in riverine ecosystems. Here, we investigated the microeukaryotic generalists and specialists along a subtropical river. Our results showed that specialists were more controlled by deterministic processes compared with generalists. Co-occurrence network analysis revealed that microeukaryotic specialists were more interconnected and contributed more to maintaining network stability. More importantly, microeukaryotic specialists also played disproportionately important roles in maintaining nutrient cycling. Overall, our results elucidated the different ecological responses of microeukaryotic generalists and specialists and emphasized the important roles of microeukaryotic specialists in maintaining ecosystem stability and functions, thus providing a new perspective to better understand the significance of specialists in riverine ecosystems.

**IMPORTANCE:**

The underlying community assembly mechanisms and biodiversity-nutrient cycling (BNC) relationships of microeukaryotic generalists and specialists remain unknown in riverine ecosystems. Here, we found that microeukaryotic habitat specialists, compared with generalists, exhibited stronger deterministic processes and contributed more to maintain co-occurrence network stability, suggesting that specialist communities were more vulnerable to environmental disturbances than generalists. Furthermore, we found specialists exhibited stronger BNC relationships, indicating specialists with narrow distribution seem to have greater capacity to support ecosystem functions. Overall, this study expands the current understanding of assembly mechanisms and BNC relationships in microeukaryotic generalists and specialists.

## INTRODUCTION

Aquatic environments, being among the most diverse ecosystems on earth, are crucial in maintaining global aquatic biodiversity and providing essential ecological services (1). River ecosystems, as an important carrier of the water cycle, play an irreplaceable role in maintaining biogeochemical cycles (2). Revealing the dynamic characteristics of river ecosystem components can improve our understanding of ecological processes in aquatic environments. Microeukaryotes are a prominent part of riverine ecosystems and perform many important ecosystem functions, including nutrient cycling and pollutant degradation (3). In addition, microeukaryotes serve as pivotal primary producers and are the principal consumers of bacterial biomass, constituting an essential component of aquatic food webs (4, 5). Recent studies have shown that various global change factors can severely impact ecosystem functions by regulating biodiversity (6, 7). Given that the community composition of eukaryotes responds quickly to environmental changes, these changes may directly affect aquatic ecosystem functions or indirectly regulate ecosystem functions through cascading effects. Therefore, a comprehensive understanding of the factors driving microeukaryotic community is essential for maintaining river ecosystem functions.

Understanding the mechanisms underlying biodiversity maintenance is a central topic in ecological research (8). Niche theory and neutral theory offer complementary frameworks for understanding microbial community assembly. The niche theory asserts that microbial communities are primarily governed by deterministic processes, including environmental filtering and biological interactions, whereas the neutral theory argues that stochastic processes (birth, death, immigration and emigration, spatiotemporal variation, and historical contingency) dominate community assembly (9). The general consensus now is that community assembly is collectively governed by both deterministic and stochastic processes, with their relative importance varying across ecological contexts. Microeukaryotes can be classified into generalists and specialists based on their ability to adapt to different ecological niches (10). Generally, generalists tend to exhibit wide niche breadth and strong environmental tolerance, leading to wide distributions. In contrast, specialists with narrow niche breadth are sensitive to environmental changes and exhibit limited distributions (10). These differences in ecological niches may lead to distinct community assembly processes between the two groups. However, the research on microeukaryotes has typically examined community assembly at the entire community level, which may not fully capture how their diversity responds to environmental disturbances (11–13). Based on the ecological characteristics of generalists and specialists, we hypothesize that the community assembly of microeukaryotic specialists is more influenced by deterministic processes compared with generalists.

Microbes are not solitary individuals, and they are embedded in complex microbial communities, interacting with a multitude of other microorganisms (14). The co-occurrence networks can be used to explore microbial interactions. Generalists appear to be easier to detect in the environment due to their wide distribution; thus, they are thought to be more likely to associate with other species and contribute significantly to maintaining community stability (15). Specialists are overwhelmingly dominant in diversity (16). A recent study has begun to focus on the specialists and revealed that specialists played an important role in maintaining interspecies interactions and network stability in urban park ecosystems (17). In fact, specialists exhibit high diversity and functional specialization. They rely on other community members for essential resources while maintaining competitive advantages in their preferred niches, thereby playing crucial roles in supporting ecosystem stability (15, 18). Therefore, we propose our second hypothesis: compared with generalists, microeukaryotic specialists are more interconnected and play an important role in maintaining network stability.

Ecosystems can provide multiple functions and services simultaneously, with nutrient cycling playing a central role as a key ecological process in maintaining ecosystem structure and functions (19). The efficiency of nutrient cycling directly determines the biomass of primary producers in aquatic ecosystems, which in turn affects the entire ecosystem’s function through the cascading effects of the food web (20). In addition, when nutrients such as nitrogen and phosphorus exceed critical ecological thresholds, it can lead to excessive algal growth, resulting in eutrophication of the rivers, which poses a significant threat to the ecological health of the aquatic ecosystems (21). Therefore, studying the biogeochemical cycling processes in aquatic ecosystems is essential for their scientific management. Microeukaryotes, such as protozoa and microalgae, are key drivers of biogeochemical cycling processes in aquatic ecosystems. These microorganisms regulate the material flow and energy transfer within the ecosystem by participating in the cycling processes of elements such as carbon (C) and nitrogen (N) (22, 23). The multi-nutrient cycling index has been used to explore the biodiversity-nutrient cycling relationship in terrestrial ecosystems (24–26). However, the relationship between microeukaryotic diversity and multi-nutrient cycling in river ecosystems remains largely unexplored. In addition, many studies have initiated with entire taxonomic groups, presuming *a priori* that different groups are functionally equivalent. In fact, specialists exhibit higher resource-use efficiency within their narrow ecological niches and can perform their functions effectively under stable environmental conditions (27). Thus, our third hypothesis is that, compared with generalists, microeukaryotic specialists with higher diversity may play stronger roles in maintaining multi-nutrient cycling.

To test these hypotheses, we employed 18S rRNA gene high-throughput sequencing to investigate microeukaryotes in a subtropical river. We analyzed the community assembly, co-occurrence network patterns, and the BNC relationship of microeukaryotic generalists and specialists. This study aimed to answer the following key questions: (i) whether microeukaryotic specialists are more influenced by deterministic processes compared with generalists, (ii) whether habitat specialists exhibit stronger interspecific interactions and contribute more to maintaining network stability, and (iii) what is the difference in the BNC relationship between generalists and specialists?

## MATERIALS AND METHODS

### Sample collection and environmental information

Tingjiang River (116°19′–116°37′E, 24°59′–25°24′N) is the largest river in the west of Fujian province, southeastern China. Along the flow direction of the river, 30 sampling sites (0.5 m depth) were selected in July (wet season) and November (dry season) 2015, respectively, resulting in 60 samples in total. The samples were collected within 5 days each season and promptly delivered to the laboratory for immediate processing. To obtain microeukaryotic plankton communities, water samples were initially filtered through a 200 µm mesh to eliminate debris and larger plankton. Subsequently, approximately 500 mL of water was filtered using a 0.22 µm pore-size polycarbonate membrane (47 mm diameter, Millipore, Billerica, MA, USA) to capture microeukaryotes.

The latitude and longitude of the sampling sites were measured by portable global positioning system (GPS Jisibao G330, Beijing, China). A total of 24 environmental factors were measured according to standard methods, including water temperature, dissolved oxygen (DO), electrical conductivity (EC), turbidity, pH, flow velocity, suspended solids, oxidation reduction potential (ORP), chlorophyll-*a* (Chl-*a*), total carbon (TC), total organic carbon (TOC), total nitrogen (TN), total phosphorus (TP), ammonium nitrogen (NH_4_-N), nitrite and nitrate nitrogen (NO_x_-N), phosphate phosphorus (PO_4_-P), arsenic (As), and heavy metals (Cr, Cu, Zn, Cd, Hg, and Pb), as previously described (28).

### DNA extraction and amplicon sequencing

The DNA of microeukaryotes was extracted using a FastDNA spin kit (MP, Biomedicals, Santa Ana, CA, USA) following the manufacturer’s instructions, and polymerase chain reaction assays were performed targeting the V9 region of the eukaryotic 18S rRNA gene using a universal primer, specifically the forward primer 1380F and reverse primer 1510R (29). Three PCR replicates for each of 60 samples were mixed and purified to reduce PCR bias using GeneJET Gel Extraction Kit (Thermo Scientific, Hudson, NH, USA). The sequencing libraries were prepared utilizing the NEB Next Ultra DNA Library Prep Kit for Illumina (New England Biolabs, Beverly, MA, USA) in accordance with the manufacturer’s protocol and finally sequenced on an Illumina platform (Illumina Inc., San Diego, CA, USA).

### Bioinformatics

Sequenced paired-end reads were merged with FLASH (30) and analyzed using QIIME v.1.8.0 to remove low-quality reads (31). Chimera sequences were identified and removed using UCHIME (32). Then, we used UPARSE with the 97% sequences similarity cutoff to cluster sequences into OTUs. After that, representative sequences from each OTU were aligned against the SILVA (Release 123) reference alignment using the RDP classifier (33). To ensure accuracy and compatibility, taxonomic assignments were validated in accordance with eukaryotic reference standards (34). Finally, to facilitate the comparison of community patterns among multiple samples, we normalized the sequences data to 110,394 sequences per sample (35).

### Statistical analyses

#### Identification of habitat generalists and specialists

Habitat generalists and specialists were defined based on niche breadth, which identified different levels of species specialization (36). Niche breadth was calculated using the formula given below for each OTU:


$$B_{j}=\frac{1}{\sum_{i=1}^{N}P_{ij}^{2}}$$

where *B*_*j*_ indicates the niche breadth of OTU *j*; N is the total number of communities; and *P*_*ij*_ is the proportion of OTU *j* in community *i* (37). A higher *B* value of OTUs indicates that the OTUs were present and more evenly distributed on a large scale (38). *B*_*j*_ can be greater than or less than the expected degree of niche breadth, indicating a broader or narrower range of habitats than expected (36, 38). Since low-abundance taxa may be temporarily absent or below detection limits in some environments, they could erroneously appear as specialists when using Levin’s niche breadth. Therefore, OTUs with mean relative abundance <2 × 10^−5^ were not considered (38). Further, to quantify the degree to which observed niche values deviated from expectations, the original OTU relative abundance table was randomly permuted 1,000 times using the "EcolUtils" package (https://github.com/GuillemSalazar/EcolUtils) (39). Each time a permutation was performed, the niche breadth for each OTU was calculated to generate a null distribution. Then, the observed niche values were compared to assess if they significantly deviate from the null distribution. OTUs were classified as generalists and specialists when the observed niche values exceeded the upper or fell below the lower 95% confidence intervals of the null distribution, and OTUs within the 95% confidence intervals of the null distribution were considered opportunists (40). A total of 275 generalists, 2,011 specialists, and 1,614 opportunists were identified (Table S1). This approach does not require setting a specific niche breadth value, thereby avoiding potential biases introduced by the arbitrary definition of the niche breadth index (38).

#### Community composition and diversity analyses

The relative abundance of microbial taxa was calculated by normalizing OTU read counts to the total reads per sample. The top 10 most abundant taxa were selected and visualized to reflect the community composition of generalists and specialists. The alpha diversity index (e.g., Richness, Chao1, and ACE) of the microeukaryotic generalists and specialists was calculated using the "vegan" package (https://cran.r-project.org/web/packages/vegan/index.html). The Wilcoxon rank-sum test was used to compare the differences in alpha-diversity between generalists and specialists (41). The beta-diversity of microeukaryotic generalists and specialists was estimated based on Bray-Curtis dissimilarity between samples. Subsequently, the nonmetric multidimensional scaling (NMDS) based on community Bray-Curtis similarity was conducted to visualize differences in the community composition of generalists and specialists between wet and dry seasons using the "vegan" package. The permutational multivariate analysis of variance (PERMANOVA) was used to evaluate the significant differences between groups using the "vegan" package.

#### Environmental drivers and distance-decay relationship analyses

To assess the impact of environmental factors (e.g., TOC, TP, and pH) on the community composition of microeukaryotic generalists and specialists, we performed constrained analysis of principal coordinates (CAP) using the "vegan" package. To explore the spatial predictor of microeukaryotic generalists and specialists, here we used dendritic network length (km), which is a measure of the cumulative length of the branching river network (watercourse) of two sampling sites to characterize the spatial distance. The geographical measures were calculated using ArcGIS (ESRI, Redlands, CA, USA). The ordinary least-squares regression was used to determine the relationships among the Bray-Curtis similarity of microeukaryotic community, the Euclidean distance of environmental factors, and the geographical distance of sampling sites.

#### Community assembly mechanism

To assess the relative importance of stochasticity and determinism for microeukaryotic community assembly, we first calculated the normalized stochasticity ratio based on the "NST" package (https://github.com/DaliangNing/NST) using generalist and specialist community data sets in wet and dry seasons (42). The stochasticity cutoff (50%) is used to quantify the balance between their relative contributions. An NST <50% suggests a higher determinism, while NST >50% suggests increased stochastic community assembly (42). Then, the null model analysis was performed using the framework described by Stegen et al. (43), which categorizes ecological processes into deterministic processes (homogeneous selection and heterogeneous selection) and stochastic processes (dispersal limitation, homogenizing dispersal, and undominated). The null model expectation was derived through 999 randomizations. Phylogenetic and taxonomic diversity variations were assessed using null model-based β-diversity metrics, specifically the β-nearest taxon index (βNTI) and the Bray-Curtis-based Raup-Crick (RC_Bray_). The βNTI value >2 or < −2 is denoted as heterogeneous selection or homogeneous selection, respectively. In contrast, |βNTI| < 2 suggests the dominance of stochastic processes. The RC_Bray_ was then applied to further assess the stochastic processes. The community assembly is dominated by dispersal limitation or homogenizing dispersal if the RC_Bray_ >0.95 or <−0.95, respectively. The RC_Bray_ between −0.95 and 0.95 is categorized as undominated (43). The above calculations were performed using the "picante" package in R (44). Mantel correlogram was used to estimate whether notable phylogenetic signals emerge at short phylogenetic distances along the environmental gradients using the "microeco" package (https://github.com/ChiLiubio/microeco).

#### Co-occurrence network construction

To estimate species coexistence across different seasons and groups, metacommunity co-occurrence networks consisting of all members of the generalists and specialists were constructed using the "igraph" package (https://github.com/igraph/igraph). To reduce rare OTUs in the data set, only OTUs' frequency of occurrence >80% and only OTUs with average relative abundance >0.05% were retained. Robust correlations (Spearman's correlation coefficients, r > 0.6) and false discovery rate (FDR)-corrected *P* < 0.05 were used to construct generalist and specialist networks. Each node corresponds to a specific OTU, and edges indicate significant correlation between two nodes. To describe the topology of the networks, we calculated a set of metrics: average path length (the average network distance between all pairs of nodes); network diameter (the greatest distance between the nodes that exist in the network); network density (the average connections of each node with another unique node in the network); degree (number of edges connecting a node to other nodes); betweenness (the number of times a node acts as a bridge along the shortest path between two other nodes) (45, 46). The average degree (avgK) represents the average number of edges per node, which represents the network complexity. Subnetwork analyses of generalist and specialist communities in wet and dry seasons were performed separately. Networks were visualized using the interactive Gephi platform (https://gephi.org/) (47).

Network stability of generalists and specialists was evaluated by natural connectivity using the "igraph" package and cohesion index (48, 49). The cohesion index includes positive cohesion and negative cohesion, which can quantify the positive and negative connectivity among taxa within a given community:


$$C_{j}^{pos}=\sum_{i=1}^{n}a_{i}\cdot {\bar{r}}_{i,r>0}(\text{ Positive cohesion })$$

and


$$C_{j}^{neg}=\sum_{i=1}^{n}a_{i}\cdot {\bar{r}}_{i,r<0}(\text{ Negative cohesion })$$

where *a*_*i*_ represents the abundance of taxon *i* in sample *j*, and $\overset{-}{r}$_*i,r* > 0_ and $\overset{-}{r}$_*i,r* < 0_ denote positive and negative connectivity, respectively (48). A network is more stable if it exhibits higher natural connectivity and ratio of absolute negative-to-positive cohesion (11). The Wilcoxon rank-sum test was used to assess significant differences between generalists and specialists for some network parameters (such as degree, betweenness, and cohesion).

To assess the ecological role of each node, two key topological metrics were integrated: the within-module connectivity (Zi), which quantifies a node’s connectivity within its own module, and the among-module connectivity (Pi), reflecting its linkages across different modules (50). Specifically, based on within-module and among-module connectivity, different nodes can be divided into four categories: (i) network hubs (Zi >2.5, Pi >0.62); (ii) module hubs (Zi >2.5, Pi ≤0.62); (iii) connectors (Zi ≤2.5, Pi >0.62); and (iv) peripherals (Zi ≤2.5, Pi ≤0.62) (50). Excluding peripheral nodes, all other nodes in each network were identified as potential keystone species that play important roles in maintaining network connectivity and stability (51). Sequentially, after identifying the keystone species, we removed these keystone species from the entire network and observed whether the natural connectivity decreases rapidly to verify their importance (52, 53). Furthermore, changes in natural connectivity were calculated by removing the generalists, specialists, and opportunists from the entire network to further assess the roles of different taxa in supporting network stability.

#### Multi-nutrient cycling index measurements

The multi-nutrient cycling index was utilized to quantify the cycling dynamics of multiple nutrients in aquatic ecosystems (54). This methodological approach aligns conceptually with the multifunctionality index framework previously established in terrestrial ecosystem research (24, 55, 56). This composite index was calculated based on eight key nutrient parameters: total carbon (TC), total organic carbon (TOC), total nitrogen (TN), ammonium nitrogen (NH_4_-N), nitrate nitrogen (NO_3_-N), nitrite nitrogen (NO_2_-N), total phosphorus (TP), and phosphate phosphorus (PO^_4_^-P). The combination of these parameters offers a comprehensive metric for evaluating the simultaneous cycling processes of major biogeochemical elements in aquatic systems. Briefly, each variable was standardized to a range of 0 to 1 using the formula (X_raw_ - X_min_) / (X_max_ - X_min_), where X_raw_, X_min_, and X_max_ represent the raw ecosystem function, its minimum, and its maximum values across all samples, respectively (24). These standardized variables were then averaged to obtain the multi-nutrient cycling index.

#### Linking biodiversity to multi-nutrient cycling

To explore the relationship between β-diversity of generalists and specialists and multi-nutrient cycling, ordinary linear regressions were conducted. Additionally, to further compare the contributions of generalists and specialists to multi-nutrient cycling, a random forest (RF) model—a machine learning algorithm for regression and classification—was performed using the "randomForest" package (https://cran.r-project.org/web/packages/randomForest/). Specifically, all OTUs were ranked by feature importance using RF analysis across 500 iterations. The number of marker OTUs was determined through 10-fold cross-validation and repeated five times. The cross-validation error curve stabilized when 30 OTUs were retained. Therefore, the top 30 OTUs with the highest mean decrease accuracy and Gini index were selected as key predictors significantly associated with multi-nutrient cycling, respectively (57). Partial least squares-path model (PLS-PM) based on the "plspm" package (https://github.com/gastonstat/plspm) was used to assess how β-diversity of generalists and specialists mediated by environmental and spatial factors affects multi-nutrient cycling (41). The model estimated both direct effects (the immediate impact of one variable on another) and indirect effects (the influence mediated through one or more intervening variables), providing a comprehensive understanding of the relationships among variables. Goodness of fit (GOF) index was used to assess the model performance.

## RESULTS

### Community composition and diversity of generalists and specialists

**Fig 1:**
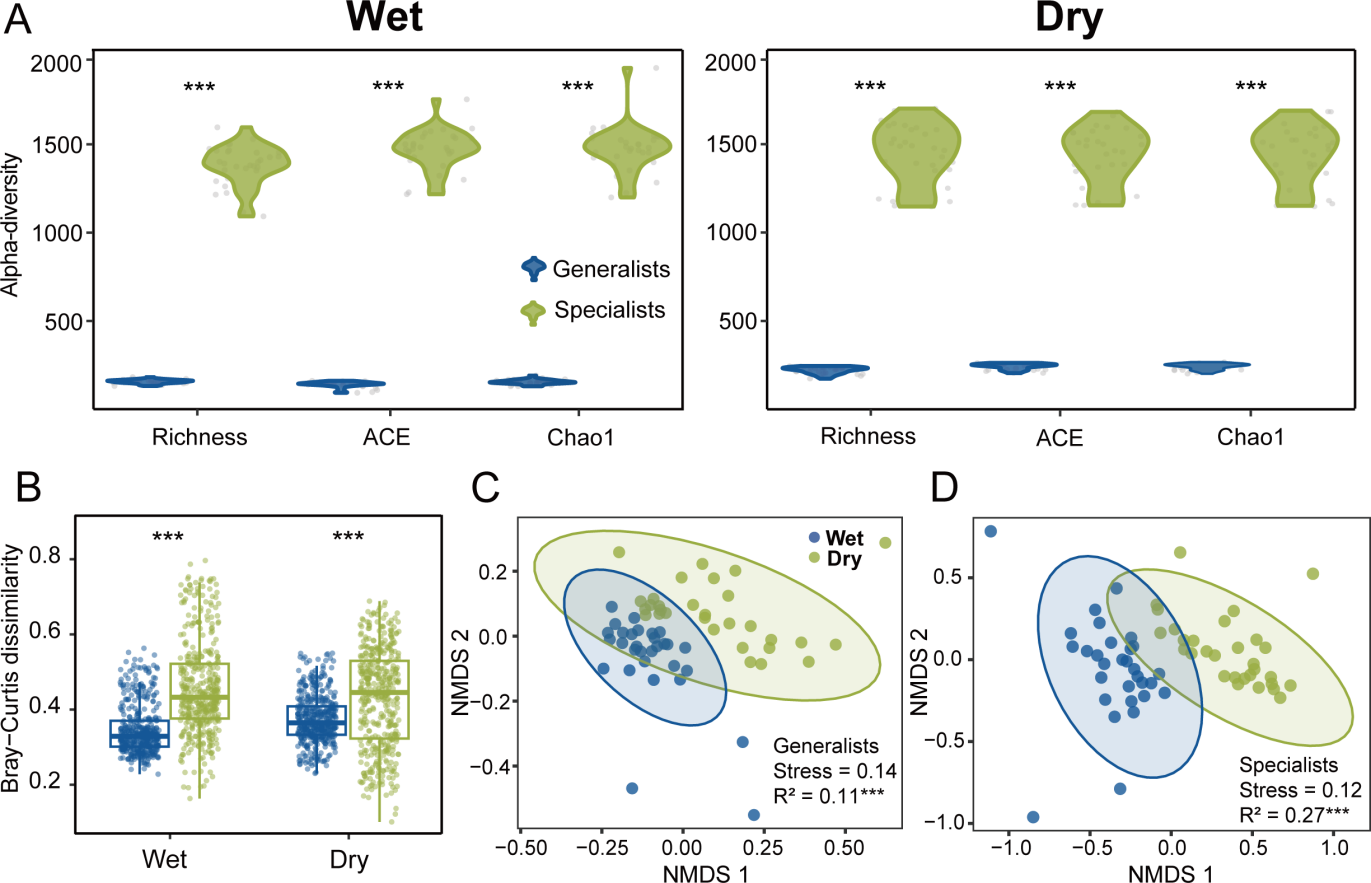
Comparison of microeukaryotic habitat generalists and specialists. (**A**) Alpha-diversity of generalists and specialists in wet and dry seasons. The data points represent the value corresponding to each sample. (**B**) Differences in beta diversity (Bray-Curtis dissimilarity) between generalists and specialists. The data points represent the Bray-Curtis dissimilarity between pairwise samples. Asterisks denote significant differences based on the Wilcoxon rank-sum test. *P < 0.05, **P < 0.01, and ***P < 0.001. (**C, D**) Non-metric multidimensional scaling analysis based on Bray-Curtis similarity showing variations in the community composition of generalists and specialists. Ellipses represent 95% confidence intervals, and data points represent the dimensionality reduction coordinates of each sample.

Taxonomic relative abundance analysis showed that the generalists maintained stable communities dominated by Stramenopiles (wet: 24.7%; dry: 26.7%), Alveolata (wet: 20.8%; dry: 19.4%), and Chloroplastida (wet: 17.4%; dry: 20.9%). In contrast, specialists shifted from dominance by Stramenopiles (wet: 31.6%) and Alveolata (wet: 21.2%) to Fungi (dry: 53.2%) and Chloroplastida (dry: 15.1%) (Fig. S1). Besides, different α and β-diversity of generalists and specialists were also observed in wet and dry seasons. The specialists showed higher α-diversity represented by Richness, ACE, and Chao1 (Fig. 1A; Table S2). Compared with generalists, the Bray-Curtis dissimilarity of specialists also was significantly higher in both wet and dry seasons, suggesting specialists exhibited higher β-diversity (Wilcoxon rank-sum test, *P* < 0.001; Fig. 1B). NMDS analysis and PERMANOVA revealed that there were significant differences in the community composition of generalists and specialists between the wet and dry seasons (*P* < 0.001; Fig. 1C and D). These differences between wet and dry seasons were larger for specialist communities than generalist communities, which suggested that the specialist communities were more influenced by seasonal variation.

### Environmental drivers and distance-decay relationship

**Fig 2:**
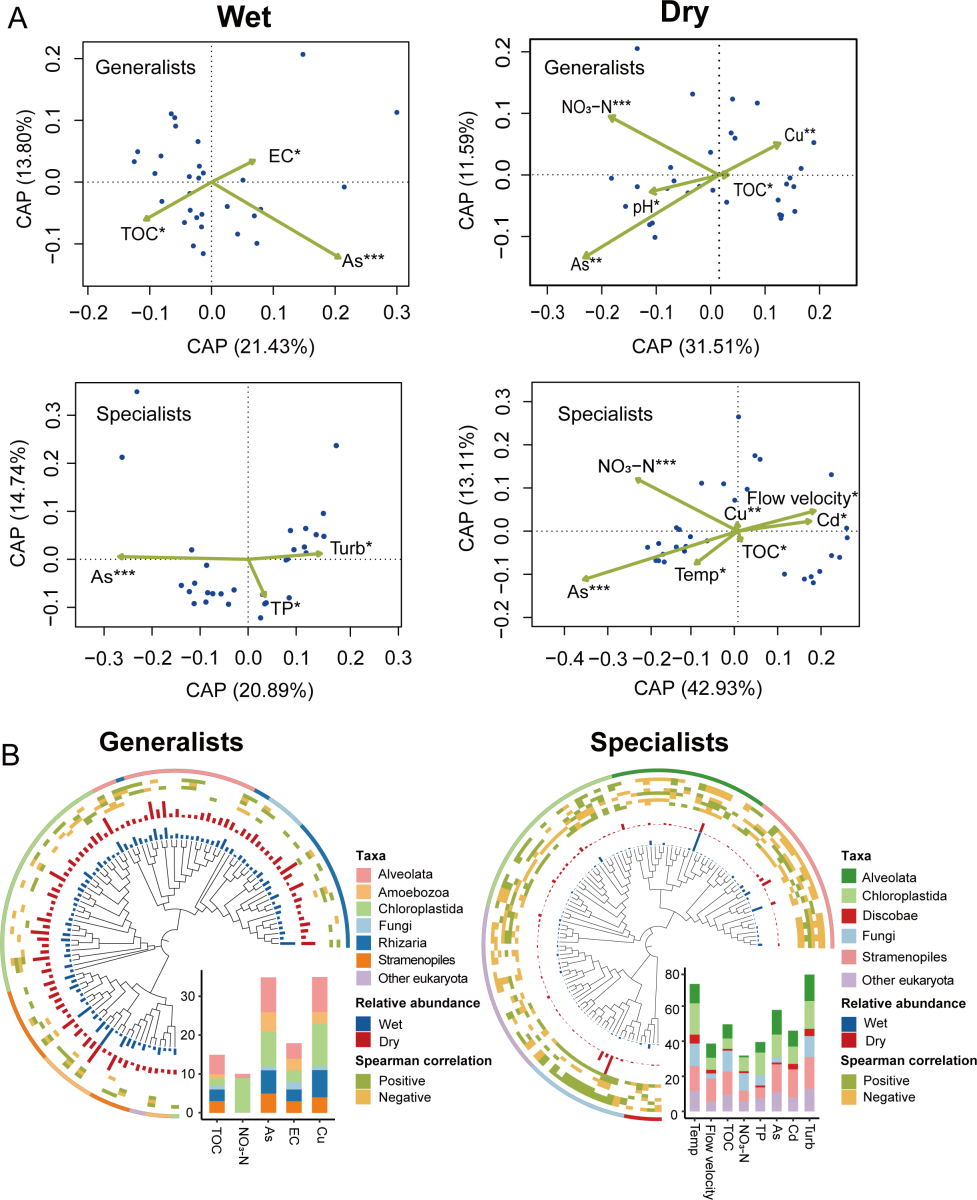
Relationship between environmental variables and microeukaryotic habitat generalists and specialists. (**A**) Constrained analysis of principal coordinates (CAP) showing environmental variables that influenced the community composition of generalists and specialists in wet and dry seasons, respectively. Statistically significant continuous variables are shown as arrows originating from the centroid of all samples. *P < 0.05, **P < 0.01, and ***P < 0.001. (**B**) Phylogeny of top 200 operational taxonomic units (OTUs) between generalists and specialists. The inner rings show the relative abundance of each OTU and their Spearman’s correlation to environmental properties (from inner to outer rings of generalists: TOC, NO3-N, As, EC, and Cu; specialists: Temp, Flow velocity, TOC, NO3-N, TP, As, Cd, and Turb). The outermost ring is the OTUs' corresponding taxa. Bar plots show the numbers of OTUs that were significantly correlated with specific environment properties.

The CAP analysis showed that the variations of generalists and specialists were determined by different environmental factors, mainly including nutrients and heavy metals (Fig. 2A). For example, TOC, EC, and As were the main drivers affecting the composition of microeukaryotic generalists, while specialists were mainly affected by As, Turb, and TP in the wet season. In the dry season, generalists were affected by NO_3_-N, pH, As, TOC, and Cu, whereas specialists were more sensitive to multiple environmental factors, including As, NO_3_-N, TOC, Cu, Cd, Temp, and Flow velocity. These findings highlighted that both generalists and specialists were more strongly influenced by environmental factors during the dry season, which explained about 43% and 55% of community variation based on CAP analysis, respectively. Phylogenetic tree analysis revealed that the relative abundance of dominant generalist taxa was higher than that of specialists and did not vary much between wet and dry seasons. In addition, dominant generalist taxa were less affected by environmental factors like heavy metals and nutrients, while dominant specialist taxa were influenced by both these factors and more physical properties of the river, such as temperature and flow velocity (Fig. 2B). This was consistent with the results of the CAP analysis (Fig. 2A).

**Fig 3:**
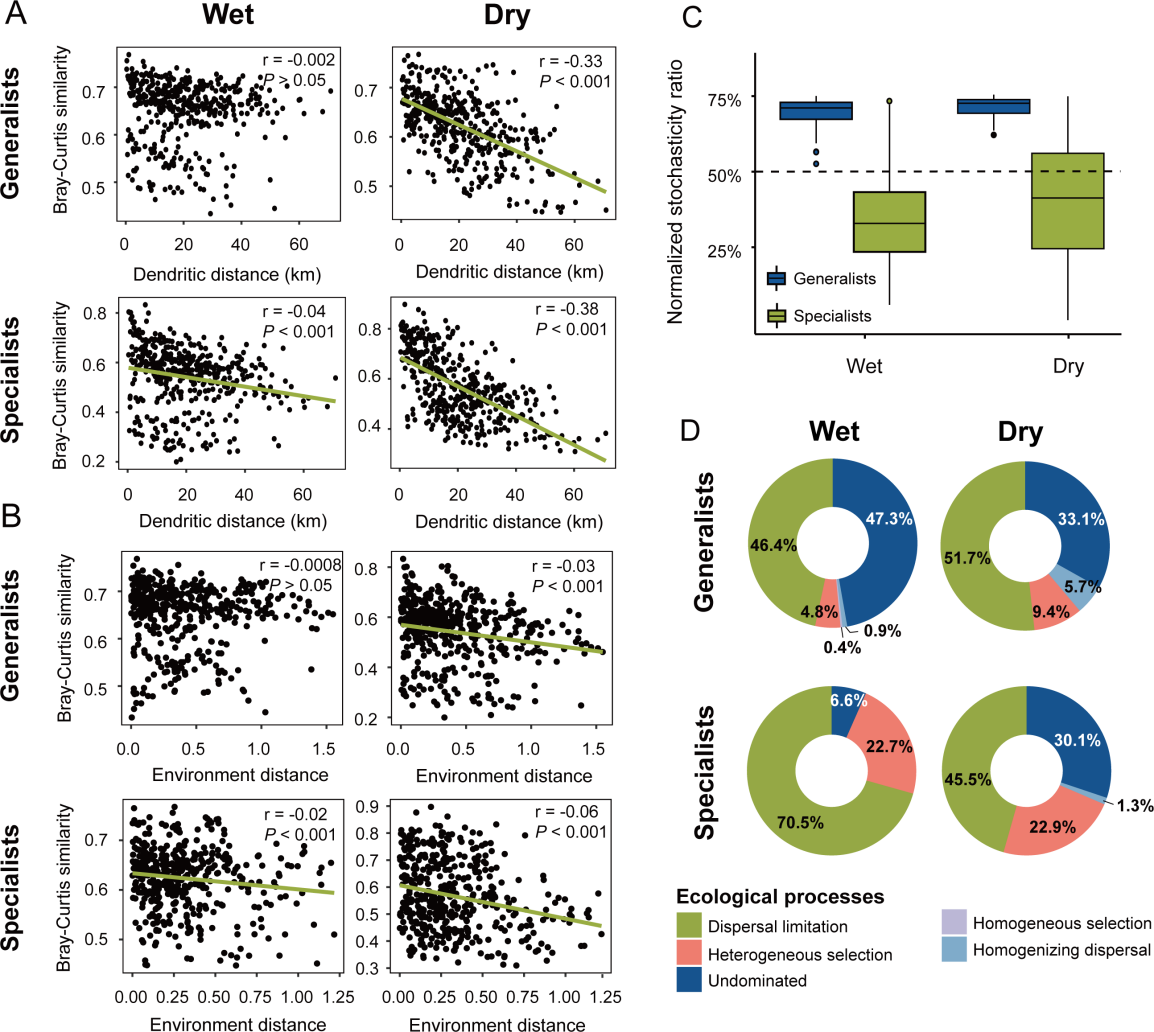
Distance decay patterns and community assembly processes of generalists and specialists. Distance-decay curves showing Bray-Curtis similarity against dendritic distance (**A**) and environmental distance (**B**) between generalists and specialists. Green lines denote the ordinary least-squares linear regressions, while the correlation coefficient (R) indicates the strength of the linear relationship. Normalized stochasticity ratio (**C**) and null model (**D**) revealed the assembly processes of generalist and specialist communities in wet and dry seasons.

To better understand the relationships between spatial and environmental distances and the community structures of microeukaryotic generalists and specialists, we conducted linear regressions of community similarity against these distances (Fig. 3A and B). In the wet season, generalists did not show a significant distance decay (*P* > 0.05); however, the specialists showed a weaker biogeographical pattern (r = −0.04, *P* < 0.001). In the dry season, both generalists and specialists showed significant distance decay patterns compared to the wet season, with specialists displaying a slightly steeper slope (r = −0.38, *P* < 0.001) than generalists (r = −0.33, *P* < 0.001) (Fig. 3A). The environmental distance-decay also revealed similar patterns. In both wet and dry seasons, the relationships between specialist communities (r = −0.02, *P* < 0.001 for the wet season; r = −0.06, *P* < 0.001 for the dry season) and environmental distance were slightly stronger than that of generalists (*P* > 0.05 for the wet season; r = −0.03, *P* < 0.001 for the dry season) (Fig. 3B).

### Community assembly processes

The relative importance of ecological processes differed significantly between microeukaryotic generalists and specialists, as evaluated by NST and null model analysis (Fig. 3C and D). A significant positive phylogenetic signal was detected at shorter phylogenetic distances, as demonstrated by the Mantel correlogram (*P* < 0.05; Fig. S2), suggesting that the community’s ecological processes can be effectively analyzed. First, the normalized stochasticity ratio (NST) values were above the 50% boundary for generalists in both wet and dry seasons, suggesting that stochastic processes played a more prominent role during the community assembly of microeukaryotic generalists. In contrast, the NST value was below the 50% boundary for specialists in the wet season and mostly below 50% in the dry season, which implied that deterministic processes were more important for the community assembly of microeukaryotic specialists (Fig. 3C). In addition, the null model result showed that the stochastic processes (including homogenizing dispersal, dispersal limitation, and undominated) explained 94.6% and 90.5% of the community assembly of microeukaryotic generalists, while they could explain 77.1% and 76.9% of the community assembly of microeukaryotic specialists in wet and dry seasons, respectively (Fig. 3D). Both the NST and null model analysis demonstrated that microeukaryotic specialists exhibited stronger determinism compared with generalists.

### Co-occurrence network analysis

**Fig 4:**
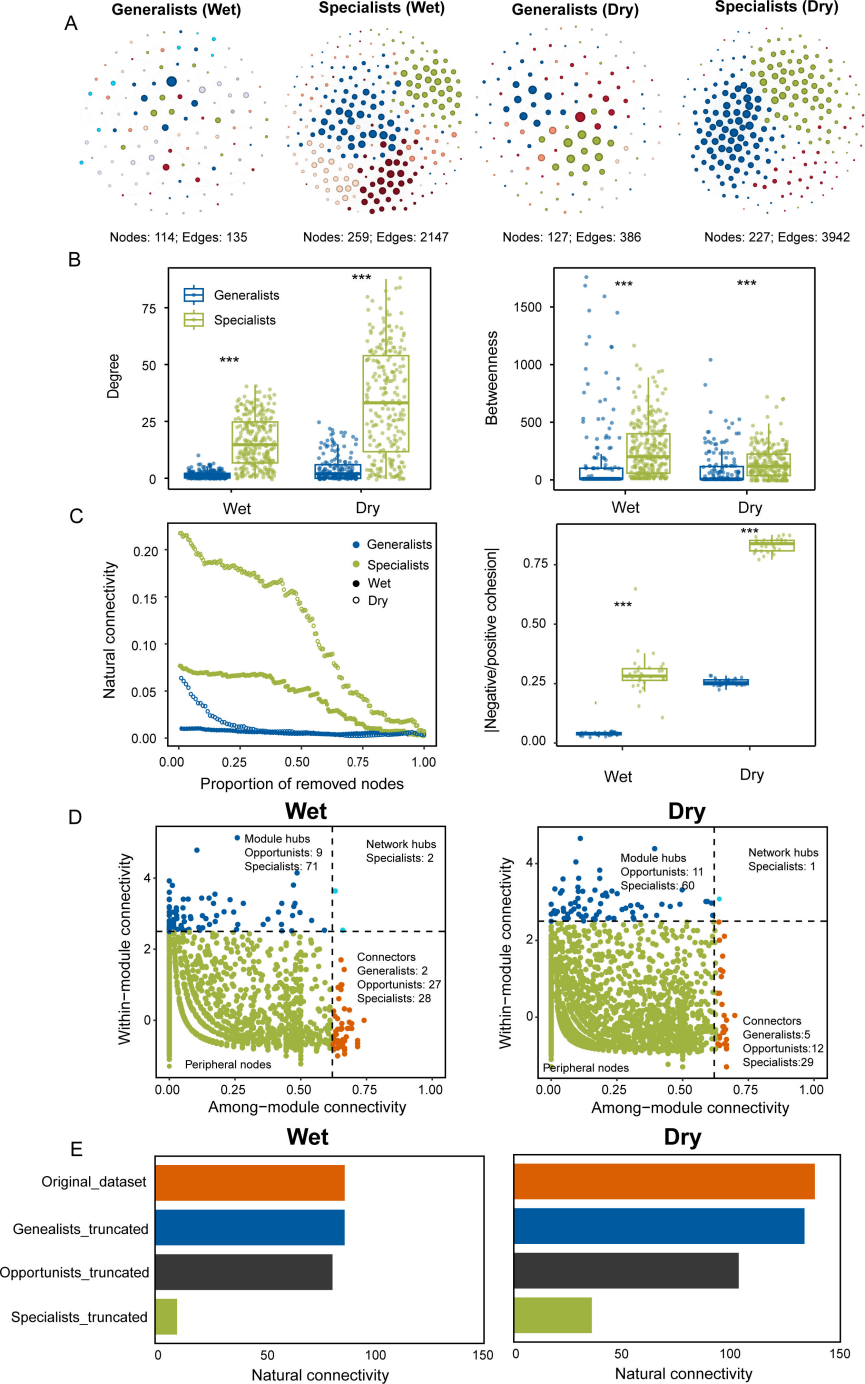
Co-occurrence network patterns of generalists and specialists. (**A**) Networks of generalists and specialists colored by modules in wet and dry seasons. (**B**) Network properties, including degree and betweenness. (**C**) Network robustness, including natural connectivity and absolute negative to positive cohesion ratio. Asterisks denote significant differences based on the Wilcoxon rank-sum test. *P < 0.05, **P < 0.01, and ***P < 0.001. (**D**) Identification of keystone species in the network based on their topological roles. Network hubs: nodes with Zi > 2.5 and Pi > 0.62; module hubs: nodes with Zi > 2.5 and Pi ≤ 0.62; connectors: nodes with Zi ≤ 2.5 and Pi > 0.62; peripheral nodes: nodes with Zi ≤ 2.5 and Pi ≤ 0.62. (**E**) The natural connectivity of the network before or after the removal of generalists / opportunists / specialists to measure their contribution to the network stability. The original data set encompassed the entire community, the generalists truncated represented the community after removing generalists, the opportunists truncated represented the community after removing opportunists, and the specialists truncated represented the community after removing specialists.

To identify potential microbial interactions, co-occurrence networks were constructed separately for microeukaryotic generalists and specialists in both wet and dry seasons, resulting in four distinct networks (Fig. 4A). Our results showed that the specialist networks exhibited shorter average path length, smaller network diameter, higher network density, and average degree than generalists (Table S3). At the node level, the specialist networks showed significantly higher degree and betweenness than generalists (Wilcoxon rank-sum test, *P* < 0.001; Fig. 4B). These results demonstrated that specialist networks were more complex and exhibited stronger interspecific interactions than generalists. Furthermore, to explore the stability of the networks for generalists and specialists, we also calculated the natural connectivity and cohesion (Fig. 4C). Compared with specialists, the natural connectivity of generalist networks decreased faster when the same proportion of nodes was removed in both wet and dry seasons. In addition, the absolute value of negative/positive cohesion of specialist networks, a property used to predict the stability of co-occurrence networks, was significantly higher than that of generalists (Wilcoxon rank-sum test, *P* < 0.001).

In order to identify the topological roles of generalist and specialist nodes, Zi-Pi plots were constructed for the entire network (including generalists, specialists, and opportunists) (Fig. 4D; Table S4). The nodes classified as network hubs, module hubs, and connectors were considered to be the potential keystone species, which have a huge impact on community structure. Notably, the removal of keystone species significantly reduced natural connectivity (slope = −0.27 in the wet season; slope = −0.35 in the dry season), compared with the random removal of species from the network (slope = −0.03 in the wet season; slope = −0.05 in the dry season) (Fig. S3). These findings supported our hypothesis that the identified keystone taxa were essential for network connectivity and probably had a pivotal role in sustaining the community stability. Overall, most of the keystone species in the network were specialists in both wet and dry seasons, indicating that specialists played important roles in maintaining the network structure. This was further supported by robustness analysis, which showed that network connectivity decreased most rapidly when specialists were removed, compared with the removal of generalists and opportunists (Fig. 4E). In summary, we demonstrated that specialist networks had stronger resistance to disturbances, which implied the importance of specialists in maintaining network structure and stability.

### Links between microbial biodiversity and multi-nutrient cycling

**Fig 5:**
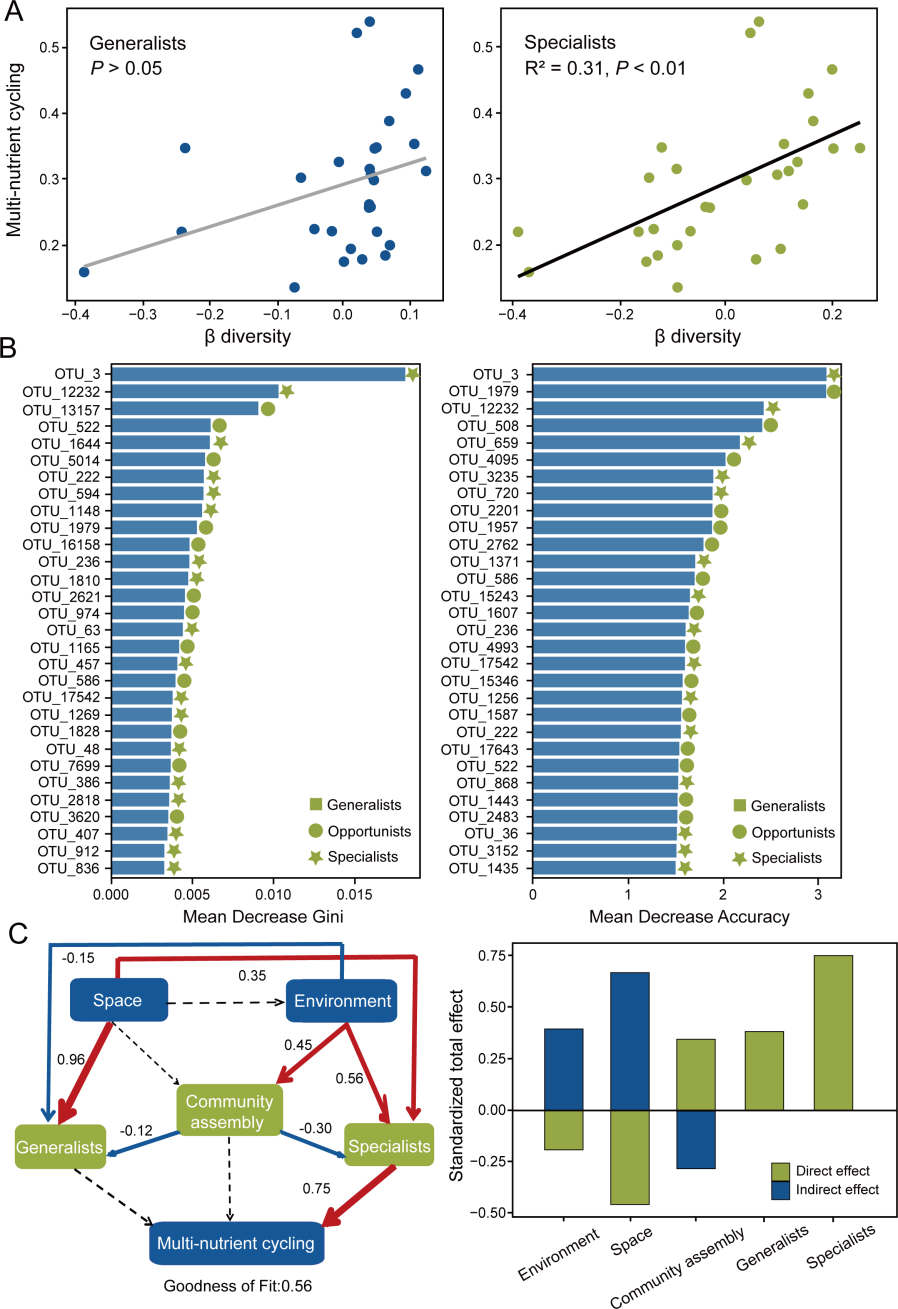
Driving factors of riverine multi-nutrient cycling. (**A**) Ordinary linear regression between multi-nutrient cycling and β-diversity of generalists and specialists in the wet season. (**B**) The mean decrease in Gini and accuracy for the top 30 OTUs based on feature importance derived from the random forest model in the wet season. (**C**) Partial least squares-path modeling (PLS-PM) showing the relationships among space, environment, community assembly, beta-diversity of generalists and specialists, and multi-nutrient cycling in the wet season. The numbers adjacent to the arrows indicate the strength of the correlations. The width of the arrows is proportional to the strength of the association. Positive relationships are denoted by red arrows, while negative relationships are represented by blue arrows. Significant relationships (*P* < 0.05) are depicted by solid arrows, whereas nonsignificant relationships are shown by dashed arrows. Goodness of fit (GOF) index was used to assess the model performance. Standardized total effects (STEs) from the PLS-PM represent the direct and indirect impacts of each variable on multi-nutrient cycling. For details of six categorized block variables and *a priori* partial least squares path models, see Fig. S5.

According to ordinary linear regression analysis, we found that microeukaryotic specialist subcommunity β-diversity contributed most to explaining the multi-nutrient cycling in the wet season, while there was no significant relationship between microeukaryotic generalist subcommunity β-diversity and multi-nutrient cycling (Fig. 5A; Fig. S4). Subsequently, to further validate whether microeukaryotic specialists contribute more to multi-nutrient cycling, we identified the top 30 predictor OTUs from all OTUs using the random forest algorithm. The results showed that specialists constituted a large proportion of the top 30 predictor OTUs, indicating that microeukaryotic specialists were important predictors for multi-nutrient cycling (Fig. 5B). A structural equation model (SEM) was subsequently constructed to quantify the contributions of the biodiversity of microeukaryotic generalists and specialists to the multi-nutrient cycling, while considering multiple drivers of multi-nutrient cycling (Fig. 5C). The multiple drivers included spatial, environmental properties, and community assembly. After considering other crucial factors, the positive and direct effects of specialist β-diversity on multi-nutrient cycling still existed. The β-diversity of generalists had no significant effect on multi-nutrient cycling.

## DISCUSSION

### Distinct patterns of microeukaryotes generalist and specialist diversity

Our results showed a few microeukaryote generalists and a significantly large number of microeukaryote specialists were detected in Tingjiang River, which indicated that the diversity of generalists and specialists was significantly different in both wet and dry seasons (Fig. 1). The generalists occupying broader niche breadth were widely distributed, but their diversity was significantly lower than that of specialists. The results can be explained by differences in ecological traits between habitat generalists and specialists. Specialists normally occupy narrow ecological niches and are susceptible to strong environmental filtering (15), leading to adaptive differentiation of species. In addition, the habitats in which they survive are quite fragmented and species are prone to geographical isolation from each other (58), leading to limited gene flow. For these reasons, they may experience more frequent species formation, which is the ultimate driver of diversification (59). However, a previous study found that habitat generalists dominated aquatic bacterial communities. The study suggested that in the context of resource substitutability, generalists may have adaptive advantages, enabling them to rapidly respond to environmental changes (60). In conclusion, our study revealed a high diversity of microeukaryotic specialists. Furthermore, we observed that the seasonal variation in specialist communities was more pronounced than that in generalist communities (Fig. 1C; D), which may be associated with the strong fluctuations in environmental conditions during wet and dry seasons of the subtropical river. This high level of environmental heterogeneity may drive significant seasonal dynamic adjustments in specialist community composition, which are more sensitive to environmental changes.

Biogeographical patterns describe the spatial and temporal distribution of species (61), while community assembly can reveal the maintenance mechanisms behind these distribution patterns (8). Here, we found that microeukaryotic specialists had slightly stronger environmental distance decay patterns than generalists in both wet and dry seasons (Fig. 3B), which may be due to specialists showing greater sensitivity when faced with environmental filtering (62). Furthermore, we found that the microeukaryotic specialists also showed slightly stronger distance-decay patterns than generalists in both wet and dry seasons (Fig. 3A). Although the magnitude of this difference was relatively small, the pattern is broadly consistent with the predictions of niche theory. Specialists, with their narrower niche breadth, are more susceptible to local extinction due to environmental filtering during dispersal. In contrast, generalists have a greater chance of moving to another site and successfully surviving in a new environment due to their broader niche width, leading to weaker biogeographical patterns (63). Notably, our study revealed significant distance-decay patterns for both microeukaryotic generalists and specialists during the dry season (Fig. 3A). This phenomenon may be closely related to the hydrological characteristics of subtropical rivers during the dry season: low-flow periods lead to a significant reduction in river connectivity, which in turn imposes stronger limitations on the dispersal of microeukaryotes, ultimately causing the two groups to exhibit more pronounced distance-decay effects (28). The seasonal hydrological dynamics regulating microbial dispersal may, to some extent, obscure the inherent niche differences between microeukaryotic generalists and specialists. Taken together, the above results implied that environmental filtering and dispersal limitation combine to shape generalist and specialist communities.

### Habitat specialists exhibit stronger determinism than generalists

Understanding community assembly mechanisms is a central issue in microbial ecology (64). Habitat generalists have greater metabolic flexibility and are favored in frequently disturbed environments, whereas specialists dominate in stable environments (63), implying that the assembly mechanisms behind the community composition of generalists and specialists differ. Here, we found that microeukaryotic specialists were affected by stronger deterministic processes compared with generalists in both wet and dry seasons (Fig. 3C and D), which were consistent with previous findings in riverine ecosystem and urban-impact ecosystems (17, 65). We ascribed this to the fact that generalists were less influenced by environmental filtering due to their broader niche breadth and were therefore more susceptible to stochastic dispersal (18). Besides, generalists displayed higher dormancy potential, a trait that promotes resilience under environmental stress and facilitates dispersal success (58). In contrast, specialists exhibited narrower niche breadth, having a high probability of extinction in random dispersal due to their poor environmental tolerance (66), thus more susceptible to deterministic processes in community assembly compared with generalists. There is evidence that at the community level, communities dominated by deterministic processes have more diverse functions than those dominated by stochastic processes (67). Under stable environmental conditions, habitat specialists contribute greatly to the stability of ecosystem functioning. However, when environmental fluctuations are frequent, generalists with high resistance to disturbance will become the backbone of ecosystem functioning. In summary, trade-offs between habitat generalists and specialists will affect ecosystem health and functions in a changing ecological context. Therefore, understanding the community assembly mechanisms behind these ecological processes is beneficial to guide the management of ecosystems.

### Habitat specialists exhibit higher co-occurrence network complexity and stability

The co-occurrence network analysis indicated that microeukaryotic specialists showed higher network complexity and stability than generalists (Fig. 4), suggesting stronger interactions among specialists. Probably, microbial generalists have larger genomes than specialists (66). Free-living organisms can evolve dependency through adaptive gene loss, which can be explained by Black Queen Hypothesis (68). This process involves two types of entities: beneficiaries (which optimize fitness by reducing the genomic complexity to alleviate metabolic burden) and helpers (which maintain larger genomes to provide public resources for the community). Generalists are more likely to be helpers due to their extensive genomic and proteomic repertoires, which enable them to utilize multiple resources, implying that interspecific interactions may not be necessary for generalists (69). Specialists may experience genome reduction in stressful environments as smaller genomes offer greater adaptive benefits, enabling more efficient resource use and lowering the metabolic burdens (70). The functions encoded by the lost genes can be gained through metabolic complementation with other community members. As a result, microbial specialists will tend to cooperate with each other in the community. Further, the network of microeukaryotic specialists also exhibited higher stability, with higher natural connectivity and absolute value of negative to positive cohesion, indicating that microbial network complexity could indeed support the stability of the microbial community network structure (71).

Ecological keystone taxa, through their metabolic roles, hold substantial importance in ecological services (72). The robustness of the network will decrease very rapidly with the removal of keystone species, suggesting that the extinction of keystone species has a dramatic impact on microbial networks (73). Here, we found that microeukaryotic specialists were crucial for preserving interactions among species and stabilizing community structure as most of the keystone taxa belonged to specialists (Fig. 4D). Therefore, the central position of specialists in the network emphasized their importance in the microeukaryotic communities (74). Our results aligned with those of a recent study, which also found that all key species belonged to specialists in urban water bodies when analyzing microeukaryotes (75). This can be explained as follows. First, the diversity of specialists and their highly specialized functions, which require cooperation with other species to obtain resources for growth, increase the likelihood of interspecies cooperation and contribute to the stability of the network. Second, although generalists have larger genomes and possess more functions, they may not be as efficient as specialists in some specific ecological niches. Thus, generalists may rely on specialists to provide more specialized functions at these ecological niches. However, the highly connected nodes can be identified by constructing microbial networks, so-called hubs. But few hub taxa have been experimentally confirmed as keystone species (76). Therefore, there is a need to strengthen the validation of experimental studies in the future.

### Habitat specialists exhibit stronger relationships between biodiversity and multi-nutrient cycling

Ecological communities often show a pattern where a low diversity of generalists coexists with a high diversity of specialists, reflecting imbalanced diversity distribution (77). Taking microeukaryotic generalists and specialists out of the whole community and evaluating their respective impacts on ecosystem functions may enhance our ability to predict microbial functional potential in aquatic ecosystems. Here, we found that the β-diversity of microeukaryotic specialists played a crucial role in supporting multi-nutrient cycling in the wet season. These findings were confirmed by different analytical methods, including ordinary linear regression and PLS-PM (Fig. 5), which confirmed the robustness of our conclusions. This result indicated that microeukaryotic specialists with narrow distribution seemed to have a greater capacity to support multi-nutrient cycling than generalists. On the one hand, in terms of their functional characteristics, specialists tend to have less functional redundancy than generalists (78). Therefore, microeukaryotic specialists may support unique nutrient cycling that is important for river ecosystem stability and health. On the other hand, microeukaryotic specialists tend to select their preferred habitats, where they can maximize the use of resources and gain a competitive advantage, and under the appropriate conditions, they may become locally dominant species, influencing multi-nutrient cycling (18, 79). As a result, increasing microeukaryotic specialist diversity accelerated the nutrient cycling process, thus enhancing the level of overall nutrient cycling in the river. In a word, our study provided new insights into the relationship between biodiversity and multi-nutrient cycling, highlighting the critical role of microeukaryotic specialists in increasing the efficiency of nutrient cycling and the stability of ecological functions from the perspective of different taxonomic groups. This view can facilitate the emergence of more purposeful and sustainable strategies to conserve the diversity of specialists and maintain the services provided by ecosystems.

Although the β-diversity of microeukaryotic generalists did not significantly affect multi-nutrient cycling in our study, it is important to note that generalists still contribute substantially to the maintenance of ecosystem functions. Generalists can occupy different ecological niches and competitively utilize a large number of resources (80) and effectively adapt to a variety of environmental changes. Therefore, under the alarming rate of biodiversity loss due to the intensification of human activities and climate change (81), microeukaryotic generalists, being highly resistant to environmental change, will play important roles in maintaining key ecosystem functions.

### Conclusion

In this study, we explored the community assembly processes, co-occurrence network patterns, and the roles of microeukaryotic generalists and specialists in supporting multi-nutrient cycling, with our conclusions aligning with the hypotheses previously proposed. We found that microeukaryotic specialists contributed significantly more to regional species diversity, exhibiting limited distribution and stronger variation in community composition between wet and dry seasons. Compared with generalists, the community assembly of microeukaryotic specialists was more influenced by deterministic processes, suggesting that the two groups exhibited different response patterns to disturbances through distinct assembly processes. In addition, microeukaryotic generalists and specialists contributed differently to maintaining species interactions, with the presence of specialists being more crucial for maintaining network stability. Furthermore, the β-diversity of microeukaryotic specialists significantly influenced multi-nutrient cycling, indicating that generalist and specialist OTUs played distinct roles in sustaining ecosystem functions. Specialists, with their high diversity and functional specificity, may play key roles in maintaining ecosystem functions. These findings enhance our understanding of the diversity and maintenance mechanisms of microeukaryotic generalists and specialists, while emphasizing the ecological importance of microeukaryotic specialists, which has significant implications for the management and protection of river ecosystem health.

## Data Availability

The DNA sequences of the 18S rRNA gene have been deposited in the National Center for Biotechnology Information (NCBI) under BioProject number PRJNA414954 and accession number SRP122256. The data that support the findings of this study are available from the corresponding author upon reasonable request.

## SUPPLEMENTAL MATERIAL

Supplemental figures aem.01364-25-s0001.docx Figures S1 to S5.

Supplemental tables aem.01364-25-s0002.xlsx Tables S1 to S4.

ASM does not own the copyrights to Supplemental Material that may be linked to, or accessed through, an article. The authors have granted ASM a non-exclusive, world-wide license to publish the Supplemental Material files. Please contact the corresponding author directly for reuse.
