# Supplementary material for: Microeukaryotic habitat specialists exhibit stronger determinism and biodiversity-nutrient cycling relationship than generalists in a subtropical river
Source: Appl Environ Microbiol. 2025 Sep 23;91(10):e01364-25. doi: 10.1128/aem.01364-25 (PMC12542682; doi:10.1128/aem.01364-25)
Supplement: Supplemental figures — Figures S1 to S5. [file aem.01364-25-s0001.docx]

*Supplementary information of the article:*

**Microeukaryotic habitat specialists exhibit stronger determinism and biodiversity-nutrient cycling relationship than generalists in a subtropical river**

Lu Li^1^, Huihuang Chen^2^, Weidong Chen^1^*, Jun Yang^2^*

^1^Key Laboratory for Humid Subtropical Eco-geographical Processes of the Ministry of Education, School of Geographical Sciences, Fujian Normal University, Fuzhou, China

^2^Aquatic EcoHealth Group, Fujian Key Laboratory of Watershed Ecology, State Key Laboratory of Regional and Urban Ecology, Institute of Urban Environment, Chinese Academy of Sciences, Xiamen 361021, China

**Running title:** Specialists exhibit stronger determinism and BNC relationship

**Keywords:** Generalists / Specialists / Community assembly / Co-occurrence network / Multi-nutrient cycling

**Subject Category:** Microbial population and community ecology

*** Corresponding author:**

E-mail address: [wd_chen@fjnu.edu.cn](mailto:wd_chen@fjnu.edu.cn) (W. Chen), [jyang@iue.ac.cn](mailto:jyang@iue.ac.cn) (J. Yang)


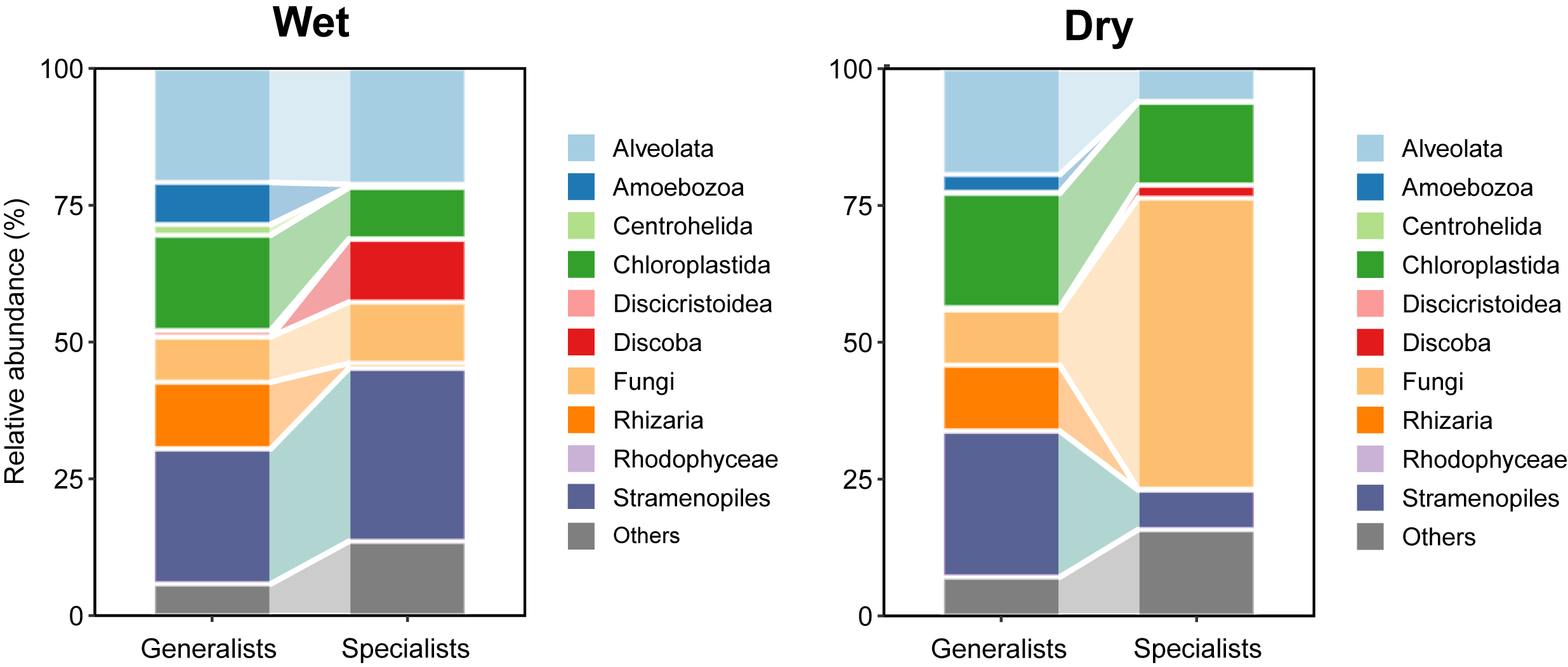


**Figure S1** Taxonomic composition of the microeukaryotic generalists and specialists in wet and dry seasons.


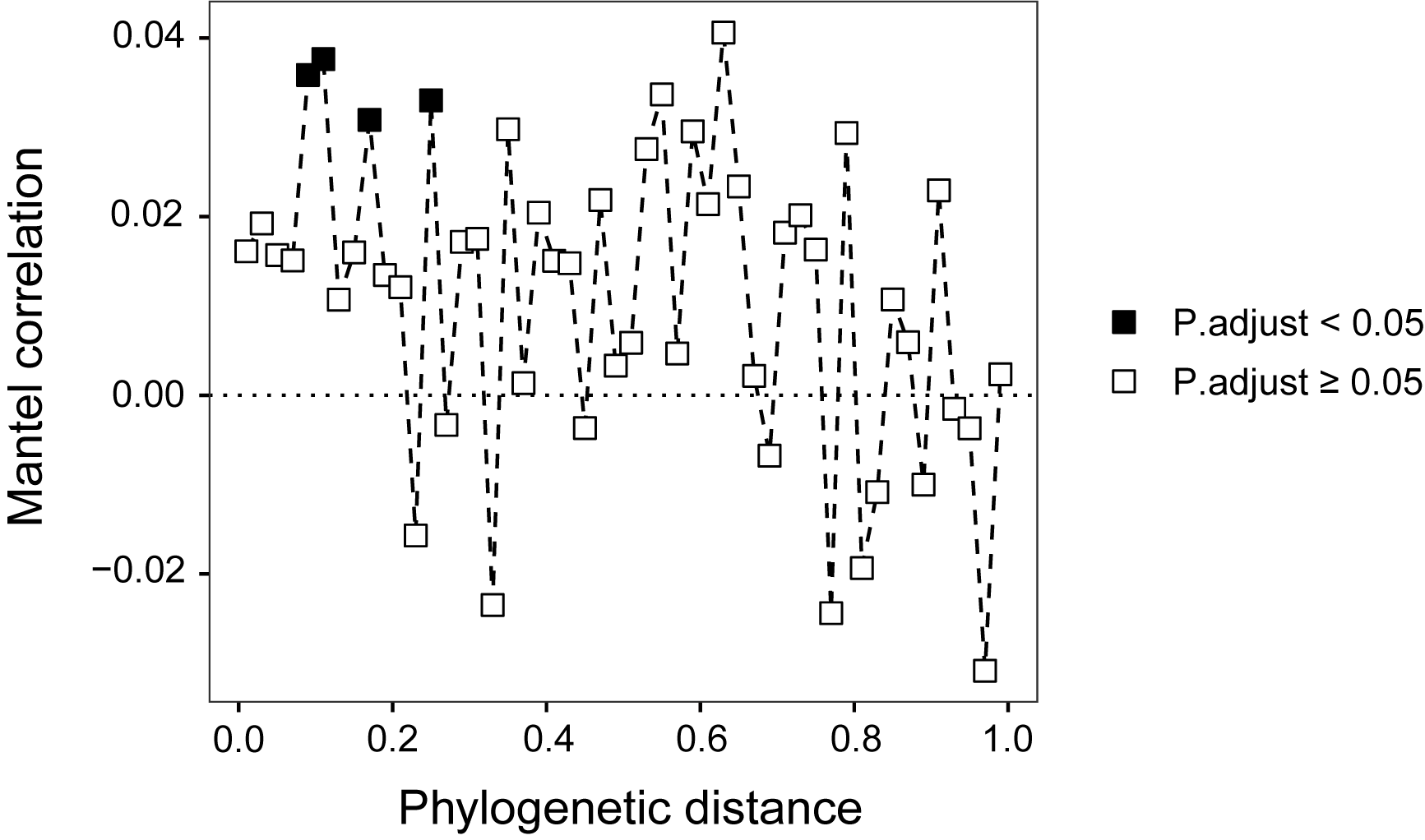


**Figure S2** Mantel correlograms signify whether notable phylogenetic signals occur at short phylogenetic distances along environmental gradients. Each point denotes the mantel correlation coefficient of the given range in phylogenetic distances. Black and white symbols separately denote significant (*P* < 0.05) and insignificant (*P* ≥ 0.05) correlations.


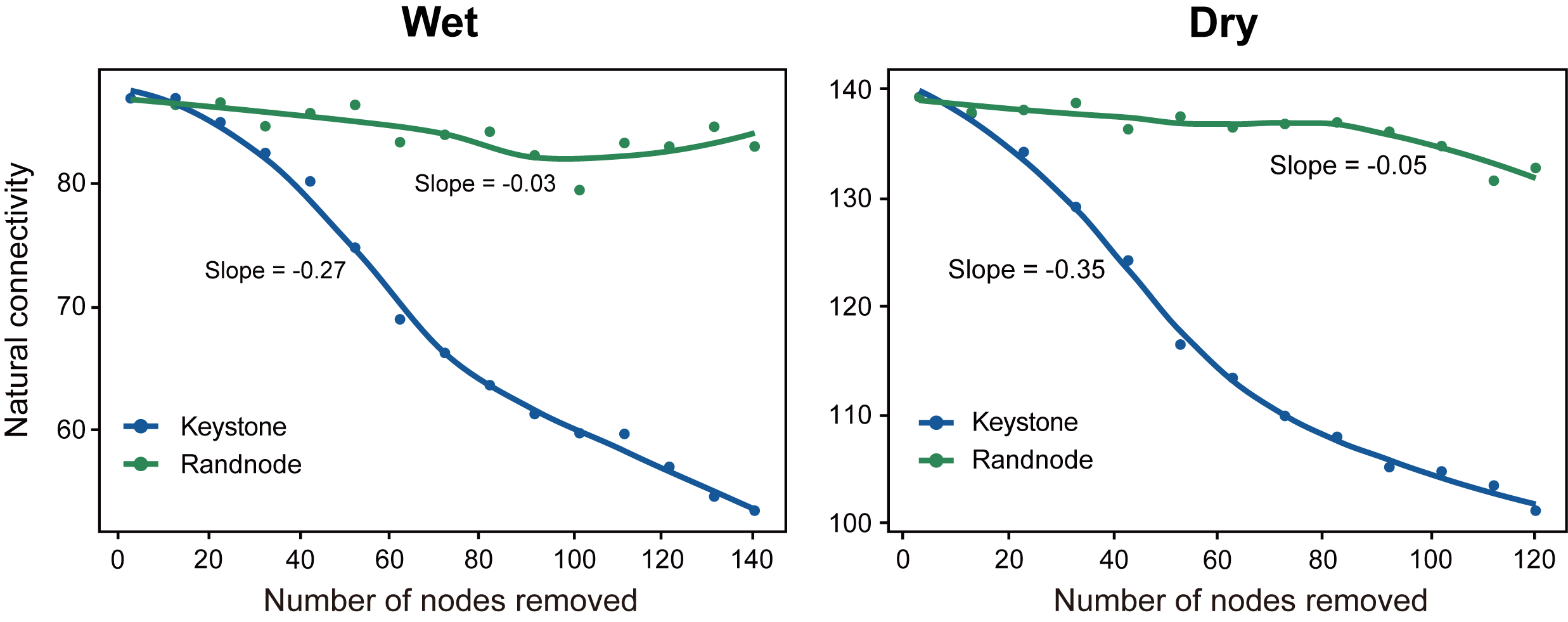


**Figure S3** The effect of targeted removal of keystone nodes and randomly removing nodes on the natural connectivity of the network. Randnode indicates a node randomly selected from the entire network. Slopes were estimated using the lm function in the stats package of R 4.3.2.


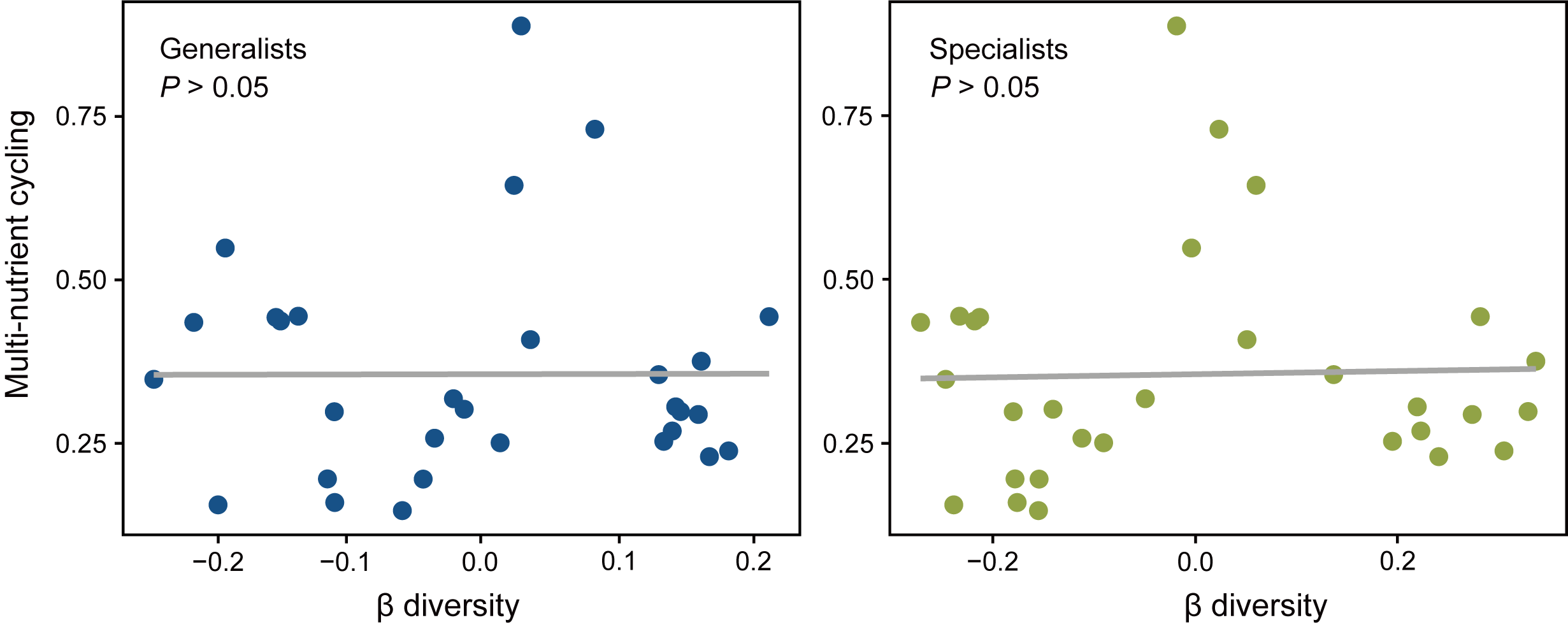


**Figure S4** The linear relationships between multi-nutrient cycling and β-diversity of generalists and specialists in dry season. Statistical analysis was performed using ordinary linear regressions.


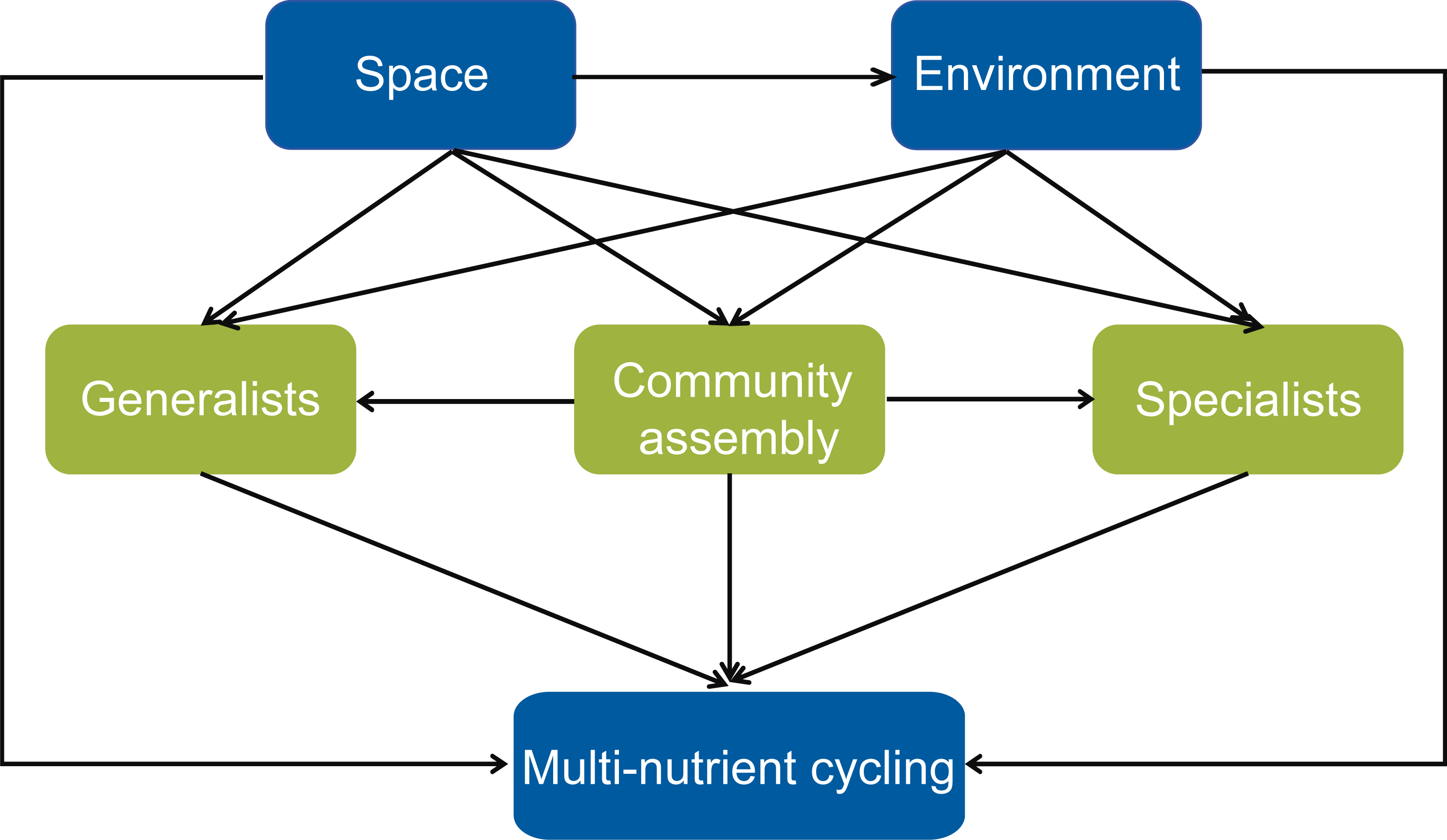


**Figure S5** *A priori* partial least squares path models (PLS-PM) aimed to evaluate the links between the beta diversity of generalists and specialists and the multi-nutrient cycling after controlling for key ecological predictors such as environment, space and community assembly in wet season. The drivers were categorized into 6 block variables: Environment (TC, TN, NO_3_-N, and NH_4_-N), Space (PCNM1), Community assembly, Generalists (beta diversity of generalists), Specialists (beta diversity of specialists), and Multi-nutrient cycling. The fitted model was available in Fig. 5C.
